# Hypolipidemic Effects of Fermented Seaweed Extracts by *Saccharomyces cerevisiae* and *Lactiplantibacillus plantarum*

**DOI:** 10.3389/fmicb.2021.772585

**Published:** 2021-11-12

**Authors:** Qiulin Yue, Zhongjian Wang, Xueyang Tang, Chen Zhao, Kunlun Li, Le Su, Song Zhang, Xin Sun, Xinli Liu, Lin Zhao

**Affiliations:** ^1^State Key Laboratory of Biobased Material and Green Papermaking, Shandong Provincial Key Laboratory of Microbial Engineering, Qilu University of Technology, Shandong Academy of Sciences, Jinan, China; ^2^Shandong Food Ferment Industry Research and Design Institute, Qilu University of Technology, Shandong Academy of Sciences, Jinan, China; ^3^Jinan Hangchen Biotechnology Co., Ltd., Jinan, China

**Keywords:** hypolipidemic, seaweed, fermentation, probiotic strains, bile acid

## Abstract

The fermentation of food materials with suitable probiotic strains is an effective way to improve biological activities. In this study, seaweed extracts were fermented by *Saccharomyces cerevisiae* and *Lactiplantibacillus plantarum*, and the hypolipidemic effects of the fermentation products were investigated. *In vitro* experiments suggested that fermented seaweed extracts have a high capacity for bile acid-binding. Additionally, a significant inhibitory effect against pancreatic lipase was observed. Furthermore, effects in hyperlipidemic mice were determined. Fermented seaweed extracts can alleviate lipid metabolism disorder. The administration of fermented seaweed extracts to mice showed decreased total cholesterol (TC), triglyceride (TG), and low-density lipoprotein cholesterol (LDL-C) levels and increased high-density lipoprotein cholesterol (HDL-C) levels. Combined, these results suggest that fermented seaweed extracts perform a potent hypolipidemic action, thus providing an effective method for the preparation of functional foods to combat cardiovascular diseases.

## Introduction

Hyperlipidemia is considered a disorder of lipid metabolism, including increased levels of total cholesterol (TC), triglyceride (TG), and low-density lipoprotein cholesterol (LDL-C), with decreased levels of high-density lipoprotein cholesterol (HDL-C; Deng et al., 2019). Abnormal lipid levels are the major contributors to the risk of cardiovascular disease causing a serious threat to human health. Due to the side effects of drug treatments for hyperlipidemia, more attention should be paid to dietary supplements to lower blood lipids.

Brown algae have attracted increasing attention because of their high growth rate and perceived health benefits. *Laminaria japonica* is one of the most well-known edible brown seaweeds widely cultivated in East Asian countries (Gao et al., 2017b). *L. japonica* is rich in polysaccharides, polyphenols, carotenoids, dietary fiber, vitamins, and minerals, and many of the compounds have been reported to exhibit a variety of biological activities (Kadam and Prabhasankar, 2010). To obtain these bioactive molecules, various methods have been applied, including acid base hydrolysis, solvent extraction, and enzymatic digestion (Tan and Lee, 2015; García-Vaquero et al., 2018). However, there are several limitations to these methods, such as high toxicity, complex procedure, and high cost. In recent years, the fermentation technique has been widely used because of its environmentally friendly processes and lower energy consumption. During fermentation, the bioactive compounds in *L. japonica* were exposed, and more importantly, the bioactivities of fermented seaweed extracts were shown to have been enhanced (Papanna et al., 2012; Kuda et al., 2015; Suraiya et al., 2018).

Several studies have reported great potential health benefits from the inclusion of a variety of probiotic strains in treatments to prevent cardiovascular disease (Hassan et al., 2019). *Lactiplantibacillus* strains showed potential to lower cholesterol in diet-induced hypercholesterolemic mice (Nami et al., 2019; Wang et al., 2019). Saikia et al. (2018) demonstrated that *Saccharomyces cerevisiae* can significantly reduce TC, LDL-C, and TG in the serum of hyperlipidemic rats. The fermentation of food materials with suitable microorganisms is a useful tool for improving the biological activities with an increased content of bioactive compound (Hur et al., 2014). Following the fermentation process, several foodstuffs, such as soybean (Pyo and Seong, 2009), cereal (Banjoko et al., 2012), and ginseng (Nan et al., 2018), have been reported to show the hypolipidemic effects. Recently, several studies have proved that combination of *L. plantarum* and *S. cerevisiae* as starter culture can improve the bioactivity (Jin et al., 2019) or flavor (Cai et al., 2020) of foods.

Hence, this study aimed to investigate the efficacy of the mixed culture of *S. cerevisiae* (AMnb091) and *L. plantarum* (LP1406) in the fermentation of *L. japonica.* Additionally, the evaluations of the hypolipidemic effect of the fermented seaweed extracts were conducted both *in vitro* and *in vivo*.

## Materials and Methods

### Materials

*Laminaria japonica* was purchased from the Tulip Crown Food Co., Ltd. (Fuzhou, China). Simvastatin, sodium cholate, sodium deoxycholate, sodium taurocholate, sodium glycocholate, cholestyramine, cellulose, orlistat, and p-nitrophenyl butyrate were purchased from Sigma-Aldrich (United States). Cellulose, pectinase, pancreatic, and pancreatic lipase were purchased from the Solarbio Technology Co., Ltd. (Beijing, China). TC, TG, LDL-C, and HDL-C kits were purchased from the Nanjing Jiancheng Bioengineering Institute (Nanjing, China). Other reagents used in the study were of analytical grade.

### Strains and Growth Condition

*Saccharomyces cerevisiae* (AMnb091) and *L. plantarum* (LP1406) were isolated and preserved in the laboratory. The *S. cerevisiae* was grown in YPD broth under shaking conditions (180 r/min) at 30°C for 24 h, whereas *L. plantarum* was grown in MRS broth at 37°C for 24 h before experimental use.

### Preparation of Fermented Seaweed Extracts

The seaweeds were washed, dried and ground, and the powder was then passed through a 40-mesh screen and collected. The seaweed powder was dissolved in distilled water at a ratio of 1:30 (w/v). Following this process, pH was adjusted to 4.8, and cellulose and pectinase were added with a dry weight of 2.5% and 0.26%, respectively. The reaction solution was stirred at 50°C for 2 h. Alkaline pectinase (0.3%) was added subsequently after pH was adjusted to 8.0, and the solution was incubated at 60°C for another 1.5 h. Finally, the reaction products were centrifuged, and the seaweed extracts were collected for fermentation.

Peptone, beef extract and glucose were added to the seaweed extracts, and pH was adjusted to 6.2. The seaweed broth was sterilized at 121°C for 20 min. Cultures of *S. cerevisiae* (AMnb091) and *L. plantarum* (LP1406) were both inoculated at a concentration of 1% (v/v) and incubated at 30°C for 2 days with shaking (180 r/min). To monitor the fermentation process, the samples were collected from the fermentations at 0 h, 12 h, 24 h, and 48 h for analyses of viable cell count and the pH value. The fermented seaweed extracts were obtained by centrifugal separation and were lyophilized. The lyophilized powders were used to evaluate the hypolipidemic effect both *in vitro* and *in vivo*. Unfermented samples were prepared using the same method without fermentation.

### Characterization of Fermented Seaweed Extracts

Moisture was measured by weighing the samples before and after overnight drying at 105°C in the oven. Ash content was measured by weighing the samples before and after ashing at 550°C for 4 h in a Muffle Furnace. Carbohydrate was evaluated via the phenol-sulfuric acid method (Wang et al., 2012), while total titratable acidity was measured according to ISO 750:1998 (International Organization for Standardization [ISO], 1998). Protein was analyzed using the Bradford method (Bradford, 1976). Amino acid content was determined with the ninhydrin assay method (Liang et al., 2005), and the soluble dietary fiber content was tested according to Association of Official Analytical Chemists (AOAC) methods 991.43 (Association of Official Analytical Chemists International [AOAC], 1990). The Folin-Ciocalteu method was used to determine total phenolics (McDonald et al., 2001).

### Bile Acid Binding Capacity Assay

The capacity of the bile acid binding of fermented seaweed extracts was measured according to the literature, with some modifications (Hamauzu and Suwannachot, 2019). Briefly, a 50 mg sample (cellulose as the negative control and cholestyramine as the positive control) was dissolved in 1 mL of 0.01 mol/L HCl to simulate gastric conditions, and then incubated at 37°C for 1 h. After adjusting the pH to 6.3, the solution was mixed with a 4 mL of 0.72 μmol/mL bile acid mixture (total 2.88 μmol of bile acids) in a 0.1 mol/L phosphate buffer and 5 mL of 10 mg/mL pancreatic (prepared by 0.1 mol/L phosphates buffer) and incubated under agitation for 1 h at 37°C. Following incubation, the mixture was centrifuged at 10,000 r/min. Free bile acid in its supernatant state was determined at 510 nm by the furfural-sulfuric acid method using a UV-Vis spectrophotometer (Hitachi U 2910) (Dziedzic et al., 2012). The bound bile acids were calculated by subtracting the amount of unbound bile acids from the added amount of bile acids. The bile acid-binding ability is expressed as μmol bile acid per gram dry matter.

### Lipase Inhibition Activity Assay

The lipase inhibition activity assay was conducted according to the literature with some modifications (Guo et al., 2018). Pancreatic lipase was prepared by using a Tris–HC1 buffer (pH 7.4) at a concentration of 5 mg/mL. Then, 100 μL of samples (orlistat as the positive control) at different concentrations of 0.5, 1.0, 10.0, 20.0, 40.0, and 50.0 mg/mL were mixed with 100 μL of an enzyme solution and 200 μL of Tris–HCl buffer. The mixture was incubated at 37°C for 10 min. Subsequently, 100 μL of the substrate solution (2 mM of p-nitrophenyl butyrate in dimethylformamide) was added into the mixture, and incubated at 37°C for 15 min. Pancreatic lipase activity was determined by measuring the absorbance of the test solution at 405 nm with a micro plate reader (MD SpectraMax i3x). The inhibition of lipase activity was expressed as the percentage decrease when pancreatic lipase was incubated with the test samples, and the IC_50_ values on pancreatic lipase were calculated by regression analysis.

### Animals and Experimental Design

Forty male C57BL/6J mice aged 5 weeks were obtained from the Junket Bioengineering Co., Ltd. (Nanjing, China). After 1 week of adaptive feeding, the mice were randomly assigned to five groups, with eight mice per group—normal control group (NC), high fat diet control group (HFC), positive control group (simvastatin, 10 mg/kg body weight/day, PC), fermented seaweed extracts group (1.2 g/kg body weight/day, FS), unfermented seaweed extracts group (1.2 g/kg body weight/day, UFS). The normal group was fed with a regular diet, while the other groups were fed with a high-fat diet that included 2% cholesterol, 10% egg yolk powder, 7% lard, and 0.5% sodium chelate. In the NC and HFC groups, the mice were administered 0.2 mL of distilled water once daily. The other groups were given 0.2 mL of daily oral administrations, respectively. The body weights were recorded weekly. After 4 weeks of different treatments, all mice were sacrificed after fasting for 12 h. Blood was taken from the eye sockets for serum lipid analysis, and the serum was frozen at −80°C. For the collection of major organ tissues, the spleen, liver, kidney, and heart, of all the mice in different groups were excised quickly and washed with a 0.9% sterilized saline solution. The organs were dried and weighed immediately. The experimental procedures were performed under the Guidance of the Regulations on the Administration of Laboratory Animals (National Science and Technology Commission of the People’s Republic of China) and approved by the Animal Ethics Committee of Shandong Province.

### Analysis of Serum Lipid Profiles

The levels of total cholesterol, triglyceride, low-density lipoprotein cholesterol, and high-density lipoprotein cholesterol were quantified using commercially developed kits (Nanjing Jiancheng Bioengineering Institute, Nanjing, China). All data were measured with a micro plate reader (MD SpectraMax i3x).

### Statistical Analysis

Results are expressed as mean ± SD. The data were analyzed using the one-way ANOVA approach with the Bonferroni test to compare differences among various groups. A value of *p* < 0.05 was considered statistically significant.

## Results and Discussion

### Microbiological Analyses of Fermented Seaweed Extracts

**FIGURE 1:**
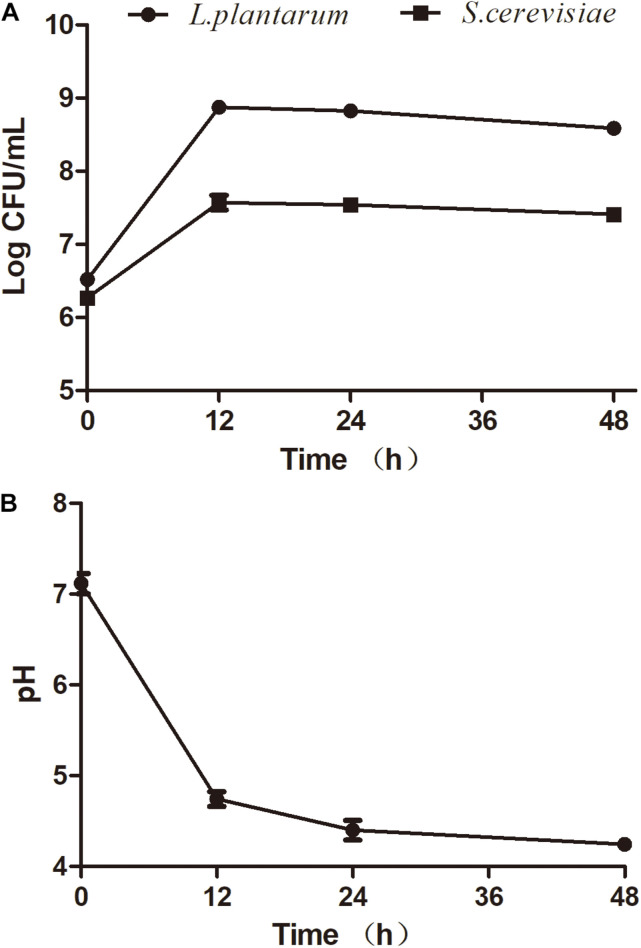
Fermentation activity of *S. cerevisiae* (AMnb091) and *L. plantarum* (LP1406) during co-culture fermentation of seaweed extracts. **(A)** Cell growth, **(B)** pH. Data are expressed in terms of means ± SD.

Several studies aimed to understand the interactions between *S. cerevisiae* and *L. plantarum* (Alexandre, 2004; Russo et al., 2020). The selection of compatible strains is a fundamental factor to ensure successful fermentation. To date, few references have been reported about the interactions between *S. cerevisiae* and *L. plantarum* for seaweed fermentation. In this study, the effect of mixed fermentations with *S. cerevisiae* (AMnb091) and *L. plantarum* (LP1406) on the seaweed extracts was investigated. The two strains exhibited similar growth behavior in the seaweed broth during the co-culture fermentation process (Figure 1A). The maximum viability of *S. cerevisiae* and *L. plantarum* was observed at 12 h after inoculations, which were 3.9 Log CFU/mL and 7.45 Log CFU/mL, respectively. However, a slight increase of cell count were detected for the two strains (12–48 h) for single fermentation, but there was no significant difference in CFU between the single and co-culture process (Figure 1A and Supplementary Figures 1A,B). This may be due to the competition for nutrients of co-culture organisms, thus inhibit each other’s growth. The results showed that the combination of the two strains can be appropriate for fermenting seaweed. A similar observation was found in the combination of *S. cerevisiae* and *L. plantarum* to ferment Mango Slurry (Jin et al., 2019). The reduction of pH value was found during fermentation (Figure 1B), and the pH decreased to 4.25 ± 0.04 at the end of the fermentation (48 h).

### Chemical Composition of Fermented Seaweed Extracts

The chemical composition of the fermented seaweed extracts and unfermented seaweed extracts are shown in Table 1. A significant decrease in carbohydrate was observed after fermentation, while the content of titratable acidity, protein, amino acids, and total phenolics was significantly increased compared with the unfermented samples. The reduced total carbohydrate was due to the utilization of sugars in seaweed extracts by the two strains. The same trend has been reported previously (Papanna et al., 2012). The higher amount of titratable acidity in the fermented seaweed extracts can be ascribed to the accumulation of organic acids during *L. plantarum* fermentation. It has been reported that increased protein and amino acid levels were detected in brown seaweed after fermentation with *S. cerevisiae*, due to the hydrolysis of polymers in the seaweed cell wall matrix (Hou et al., 2015). The results of this experiment were in accordance with that conclusion. Previous studies have showed that fermentation of brown algae can enhance the phenolic content, including yeast (Eom et al., 2011), *Aspergillus oryzae* (Rafiquzzaman et al., 2015), and red molds (Suraiya et al., 2018). Similar results were recorded in this study.

**TABLE 1:** Chemical composition of unfermented seaweed extracts and fermented seaweed extracts.

Component (%)	UFS	FS
Moisture	4.25 ± 0.008^a^	4.15 ± 0.002^a^
Ash	1.65 ± 0.001^a^	1.90 ± 0.005^a^
Carbohydrate	39.96 ± 0.003^a^	32.12 ± 0.008^b^
Titratable acidity	6.66 ± 0.001^b^	14.79 ± 0.009^a^
Protein	19.90 ± 0.006^b^	29.04 ± 0.023^a^
Amino acids	1.76 ± 0.000^b^	3.38 ± 0.000^a^
Soluble dietary fiber	3.70 ± 0.004^a^	3.30 ± 0.001^a^
Total phenolic	0.49 ± 0.001^b^	1.06 ± 0.000^a^

*Data are represented as mean ± SD from three replicates. Values in the same row with different lowercase letters represent significant differences (p < 0.05) from each other.*

### Effects of Fermented Seaweed Extracts on Bile Acid Binding Capacity

**FIGURE 2:**
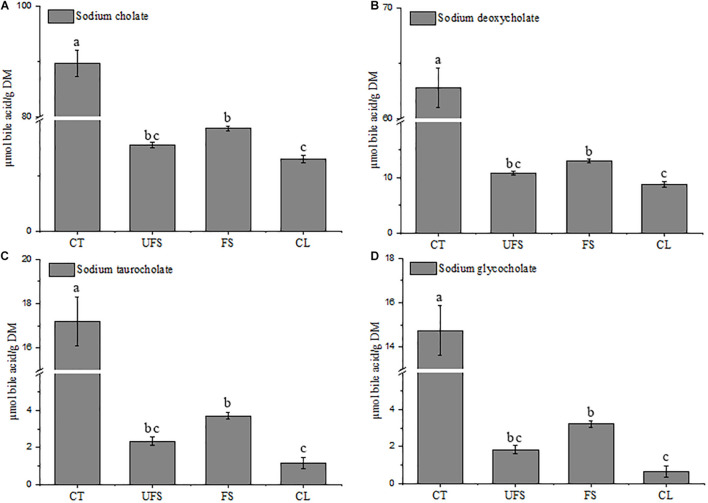
Bile acid binding capacity of unfermented seaweed extracts and fermented seaweed extracts. **(A)** Sodium cholate, **(B)** sodium deoxycholate, **(C)** sodium taurocholate, **(D)** sodium glycocholate. CT, cholestyramine; UFS, unfermented seaweed extracts; FS, fermented seaweed extracts; CL, cellulose. Data were done in triplicates and represented as mean ± SD. *p* < 0.05 is indicated by different letters.

Many studies have revealed that soluble dietary fibers or polysaccharides could reduce serum cholesterol due to their binding capacities against bile acids (Zhou et al., 2006; Gunness and Gidley, 2010; Gomez et al., 2018). Gao et al. (2017b) provided scientific evidence of how the structure characterizations of polysaccharide fractions from *L. japonica* affect the capacity of bile acid-binding. Cholic acid, glycocholic acid, and taurocholic acid are the primary bile acids synthesized in the human body (Gao et al., 2017a), and exist mainly in the form of bile salts. In this study, sodium cholate, sodium deoxycholate, sodium taurocholate, and sodium glycocholate were used to determine the capacity of bile acid-binding of fermented seaweed extracts. As shown in Figure 2, FS showed moderate activities of 18.53, 13.01, 3.72, and 3.23 μmol/g for sodium cholate, sodium deoxycholate, sodium taurocholate, and sodium glycocholate, respectively. The bile acid-binding capacities of FS were significantly higher than those of cellulose, and lower than cholestyramine. Moreover, FS had higher capacities for binding of bile acids than those from UFS group, although the difference was not significant, thus indicating the hypolipidemic potential of the fermented seaweed extracts.

### Effects of Fermented Seaweed Extracts on Lipase Inhibition Activity

Pancreatic lipase is a key enzyme for triglyceride absorption in lipid metabolism. Inhibition of lipase is considered as an effective mechanism to inhibit the absorption of triacylglycerides in patients with hypercholesterolemia (Costamagna et al., 2016; Bello et al., 2017). Thus, a lipase inhibition activity assay is widely used to evaluate the hypolipidemic effect of natural products *in vitro*, including oregano (Gutiérrez-Grijalva et al., 2018), wheat bran (Talawar et al., 2017), and brown algae (Eom et al., 2013). The inhibitory capacity of the test compounds against pancreatic lipase is shown in Table 2. The IC_50_ values of unfermented seaweed extracts, fermented seaweed extracts and orlistat were 6.14 ± 0.15 mg/mL, 3.51 ± 0.21 mg/mL, and 1.43 ± 0.05 mg/mL, respectively. FS exerted significantly stronger pancreatic lipase inhibition effect compared with UFS group, suggesting a better hypolipidemic effect of seaweed extracts after fermentation.

**TABLE 2:** Lipase inhibition activity of unfermented seaweed extracts and fermented seaweed extracts.

Group	Pancreatic lipase IC_50_ value (mg/mL)
UFS	6.14 ± 0.15^a^
FS	3.51 ± 0.21^b^
Orlistat	1.43 ± 0.05^c^

*Data were done in triplicates and represented as mean ± SD. p < 0.05 is indicated by different letters.*

### Effects of Fermented Seaweed Extracts on the Growth of High Fat Diet Mice

**FIGURE 3:**
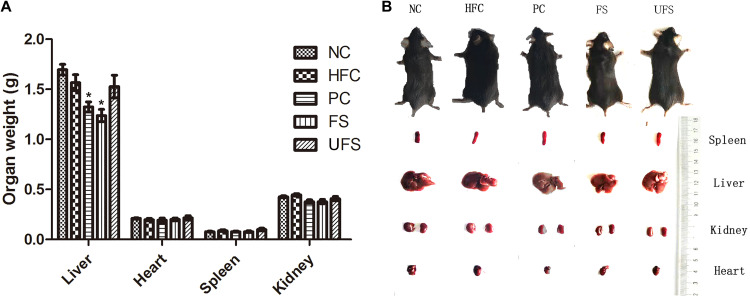
Morphologic images and weights of major organ tissues. **(A)** Organ weights in five groups of mice. Data are presented as mean ± SD (*P < 0.05). **(B)** Representative morphologic images of major organ tissues of five groups of mice. NC, normal control; HFC, high fat diet control; PC, positive control; FS, fermented seaweed extracts; UFS, unfermented seaweed extracts.

The body weight changes during the experimental period of five groups of mice are shown in Table 3. The HFC group had the highest body weight after treatment for 4 weeks. However, no group of mice showed any significant difference in body weight. Additionally, no groups showed any obvious changes in the weights of the heart, spleen, or kidney (Figure 3). The livers of the mice in the PC and FS groups were significantly lighter than those in the NC group. A similar observation was found in the hypolipidemic effects of fermented soybean (Pyo and Seong, 2009). Fermented seaweed extracts may influence hepatic cholesterol metabolism, which might lead to a lower liver weight.

**TABLE 3:** Body weight evolution during the experimental period of five groups of mice.

Group	Body weight (g)				
	0 weeks	1 weeks	2 weeks	3 weeks	4 weeks
NC	23.25 ± 0.84^a^	23.85 ± 1.14^a^	24.91 ± 1.13^a^	25.58 ± 1.04^ab^	29.65 ± 2.30^a^
HFC	25.12 ± 1.24^a^	25.99 ± 1.19^a^	27.10 ± 1.19^a^	27.47 ± 1.09^a^	30.46 ± 2.71^a^
PC	24.97 ± 1.49^a^	25.07 ± 1.98^a^	25.58 ± 1.42^a^	25.95 ± 1.53^ab^	28.12 ± 1.80^a^
FS	24.60 ± 0.65^a^	24.13 ± 0.85^a^	25.15 ± 1.60^a^	24.79 ± 1.76^b^	27.14 ± 1.54^a^
UFS	24.04 ± 0.82^a^	24.00 ± 1.61^a^	25.19 ± 1.22^a^	25.51 ± 1.28^ab^	28.69 ± 0.76^a^

*The body weight was measured once a week in each group. Data are expressed as mean ± SD for eight mice in each group. P < 0.05 is indicated by different letters.*

### Effects of Fermented Seaweed Extracts on Lipid Profiles

The changes in serum lipids of the different groups after 4 weeks of treatment are summarized in Table 4. The levels of TC, TG, and LDL-C in the HFC group were significantly higher than those of the NC group, which indicates that hyperlipidemia was successfully induced with the high-fat diet. Compared with the HFC group, the PC, FS, and UFS groups significantly decreased the serum TC, TG, and LDL-C levels and increased HDL-C levels. Significant differences existed between the FS and UFS groups for the lipid profiles, which suggest that the seaweed extracts can effectively alleviate lipid metabolism disorder after fermentation. The administration of fermented seaweed extracts to restore TC and TG to normal levels showed a similar effect to the hypolipidemic drug. More importantly, a significant decrease of serum LDL-C levels was observed in the FS group compared with the PC group. These results indicate that fermented seaweed extracts perform a potential hypolipidemic action similar to, or even better than, the hypolipidemic drug.

**TABLE 4:** Serum lipid profiles of five groups of mice.

Group	TC (mmol/L)	TG (mmol/L)	HDL-C (mmol/L)	LDL-C (mmol/L)
NC	4.21 ± 0.59^d^	2.55 ± 0.61^c^	3.31 ± 0.56^d^	0.71 ± 0.09^cd^
HFC	6.78 ± 1.36^a^	3.34 ± 0.56^a^	5.07 ± 0.69^c^	1.50 ± 0.33^a^
PC	5.11 ± 0.66^c^	2.43 ± 0.47^cd^	5.68 ± 0.49^a^	0.81 ± 0.64^c^
FS	5.28 ± 0.55^c^	2.33 ± 0.34^d^	5.51 ± 0.97^b^	0.62 ± 0.46^d^
UFS	5.72 ± 0.80^b^	2.91 ± 0.25^b^	5.11 ± 0.91^c^	0.99 ± 0.29^b^

*Data are presented as mean ± SD (n = 8). P < 0.05 is indicated by different letters.*

Studies have shown that seaweeds effectively improve abnormal lipid metabolism by the improvement of lipid profiles (Ara et al., 2002; Bocanegra et al., 2009). The hypercholesterolemia effects of probiotics on atherosclerosis also involve an alteration of LDL-C, HDL-C, and serum cholesterol levels (Hassan et al., 2019). In this study, probiotic-fermented seaweed extracts inhibited the increase of TC, TG, and LDL-C levels and increased the level of HDL-C. This research proposes that the hypolipidemic function of fermented seaweed extracts might be due to the effects of metabolites in both seaweed and probiotics. Further studies are required to investigate the active compounds in seaweed and probiotics, and the synergistic action of these components in their contribution to the hypolipidemic activity of fermented seaweed extracts.

## Conclusion

This study focused on the lipid metabolism of fermented seaweed extracts using different *in vitro* and *in vivo* methodologies. The results showed that fermentation greatly affect the levels of different phytochemicals, bile acid-binding capacity, lipase inhibition activity, and hypolipidemic effect on HFD mice. Such effects greatly strengthen the potential of this brown algae as functional food and hypolipidemic agents.

## Data Availability Statement

The original contributions presented in the study are included in the article/Supplementary Material, further inquiries can be directed to the corresponding authors.

## Author Contributions

QY and LZ were responsible for the conception of the study. QY wrote the final draft of the manuscript. XL and LZ coordinated the work and revised the manuscript. ZW carried out seaweed fermentation and *in vivo* hypolipidemic activity analysis. XT carried out *in vitro* hypolipidemic activity analysis. CZ carried out the characterization of fermented seaweed extracts. KL and LS were responsible for the data curation. SZ and XS performed the statistical analysis. All authors contributed to the article and approved the submitted version.

## Conflict of Interest

KL is employed by the Jinan Hangchen Biotechnology Co., Ltd. The remaining authors declare that the research was conducted in the absence of any commercial or financial relationships that could be construed as a potential conflict of interest.

## Publisher’s Note

All claims expressed in this article are solely those of the authors and do not necessarily represent those of their affiliated organizations, or those of the publisher, the editors and the reviewers. Any product that may be evaluated in this article, or claim that may be made by its manufacturer, is not guaranteed or endorsed by the publisher.

## Supplementary Material

Supplementary Figure 1 Cell growth of *L. plantarum* **(A)** and *S. cerevisiae* **(B)** during single culture fermentations.

## References

[B1] Alexandre H. (2004). *Saccharomyces cerevisiae*–*Oenococcus oeni* interactions in wine: current knowledge and perspectives. *Int. J. Food Microbiol.* 93 141–154. 10.1016/j.ijfoodmicro.2003.10.103 15135953

[B2] Association of Official Analytical Chemists International [AOAC] (1990). *AOAC International Methods.* Rockville: AOAC International.

[B3] Ara J. Viqar S. Qasim R. Ahmad V. (2002). Hypolipidaemic activity of seaweeds from Karachi Coast. *Phytother. Res.* 16 479–483. 10.1002/ptr.909 12203271

[B4] Banjoko I. Adeyanju M. Ademuyiwa O. Adebawo O. Olalere R. Kolawole M. (2012). Hypolipidemic effects of lactic acid bacteria fermented cereal in rats. *Lipids Health Dis.* 11:170. 10.1186/1476-511X-11-170 23231860 PMC3548745

[B5] Bello M. Lucia B.-A. Vazquez M. J. Avalos Soriano A. Correa-Basurto J. (2017). Molecular recognition between pancreatic lipase and natural and synthetic inhibitors. *Int. J. Biol.* 98 855–868. 10.1016/j.ijbiomac.2017.01.150 28212930

[B6] Bocanegra A. Bastida S. Benedí J. Nus M. Sánchez-Montero J. M. Sánchez-Muniz F. J. (2009). Effect of seaweed and cholesterol-enriched diets on postprandial lipoproteinaemia in rats. *Br. J. Nutr.* 102 1728–1739. 10.1017/S000711450999105X 19728895

[B7] Bradford M. (1976). A rapid and sensitive method for the quantitation of microgram quantities of protein utilizing the principle of protein-dye binding. *Anal. Biochem.* 72 248–254. 10.1016/0003-2697(76)90527-3 942051

[B8] Cai Z. Ruan Y. He J. Dang Y. Cao J. Sun Y. (2020). Effects of microbial fermentation on the flavor of cured duck legs. *Poult. Sci.* 99 4642–4652. 10.1016/j.psj.2020.06.019 32868009 PMC7598141

[B9] Costamagna M. S. Zampini C. Alberto M. Cuello S. Torres S. Pérez J. (2016). Polyphenols rich fraction from Geoffroea decorticans fruits flour affects key enzymes involved in metabolic syndrome, oxidative stress and inflammatory process. *Food Chem.* 190 392–402. 10.1016/j.foodchem.2015.05.068 26212988

[B10] Deng X. Ma J. Song M. Jin Y. Ji C. Ge W. (2019). Effects of products designed to modulate the gut microbiota on hyperlipidaemia. *Eur. J. Nutr.* 58 2713–2729. 10.1007/s00394-018-1821-z 30238315

[B11] Dziedzic K. Górecka D. Kucharska M. Przybylska B. (2012). Influence of technological process during buckwheat groats production on dietary fibre content and sorption of bile acids. *Food Res. Int.* 47 279–283. 10.1016/j.foodres.2011.07.020

[B12] Eom S.-H. Kang Y.-M. Park J.-H. Yu D.-U. Jeong E.-T. Lee M.-S. (2011). Enhancement of polyphenol content and antioxidant activity of brown alga *Eisenia bicyclis* extract by microbial fermentation. *J. Fish Aquat. Sci.* 14 192–197. 10.5657/FAS.2011.0192

[B13] Eom S.-H. Lee M.-S. Lee E.-W. Kim Y.-M. Kim T. (2013). Pancreatic lipase inhibitory activity of phlorotannins isolated from *Eisenia bicyclis*. *Phytother. Res.* 27 148–151. 10.1002/ptr.4694 22473750

[B14] Gao J. Lin L. Sun B. Zhao M. (2017a). A comparison study on polysaccharides extracted from: *Laminaria japonica* using different methods: structural characterization and bile acid-binding capacity. *Food Funct.* 8 3043–3052. 10.1039/C7FO00218A 28805835

[B15] Gao J. Lin L. Sun B. Zhao M. (2017b). Comparison study on polysaccharide fractions from *Laminaria japonica*: structural characterization and bile acid binding capacity. *J. Agric. Food Chem.* 65 9790–9798. 10.1021/acs.jafc.7b04033 29023123

[B16] García-Vaquero M. Rajauria G. Brijesh kumar T. Sweeney T. O’Doherty J. (2018). Extraction and yield optimisation of fucose, glucans and associated antioxidant activities from *Laminaria digitata* by applying response surface methodology to high intensity ultrasound-assisted extraction. *Mar. Drugs* 16:257. 10.3390/md16080257 30061548 PMC6117709

[B17] Gomez M. Singh J. Acharya P. Jayaprakasha G. Patil B. (2018). Identification and quantification of phytochemicals, antioxidant activity, and bile acid-binding capacity of garnet stem dandelion (*Taraxacum officinale*). *J. Food Sci.* 83 1569–1578. 10.1111/1750-3841.14169 29802721

[B18] Gunness P. Gidley M. (2010). Mechanisms underlying the cholesterol-lowering properties of soluble dietary fibre polysaccharides. *Food Funct.* 1 149–155. 10.1039/c0fo00080a 21776465

[B19] Guo H. Lin S. Lu M. Gong J. D. B. Wang L. Zhang Q. (2018). Characterization, in vitro binding properties, and inhibitory activity on pancreatic lipase of β-glucans from different Qingke (Tibetan hulless barley) cultivars. *Int. J. Biol. Macromol.* 120 2517–2522. 10.1016/j.ijbiomac.2018.09.023 30195000

[B20] Gutiérrez-Grijalva E. Antunes-Ricardo M. Acosta-Estrada B. Gutiérrez-Uribe J. Heredia J. (2018). Cellular antioxidant activity and in vitro inhibition of α-glucosidase, α-amylase and pancreatic lipase of oregano polyphenols under simulated gastrointestinal digestion. *Food Res. Int.* 116 676–686. 10.1016/j.foodres.2018.08.096 30716995

[B21] Hamauzu Y. Suwannachot J. (2019). Non-extractable polyphenols and in vitro bile acid-binding capacity of dried persimmon (*Diospyros kaki*) fruit. *Food Chem.* 293 127–133. 10.1016/j.foodchem.2019.04.092 31151592

[B22] Hassan A. Din A. U. Zhu Y. Zhang K. Li T. Wang Y. (2019). Updates in understanding the hypocholesterolemia effect of probiotics on atherosclerosis. *Appl. Microbiol. Biotechnol.* 103 5993–6006. 10.1007/s00253-019-09927-4 31201452

[B23] Hou X. Hansen J. Bjerre A. (2015). Integrated bioethanol and protein production from brown seaweed *Laminaria digitata*. *Bioresour. Technol.* 197 310–317. 10.1016/j.biortech.2015.08.091 26342344

[B24] Hur S. J. Lee S. Y. Kim Y.-C. Choi I. Kim G.-B. (2014). Effect of fermentation on the antioxidant activity in plant-based foods. *Food Chem.* 160 346–356. 10.1016/j.foodchem.2014.03.112 24799248

[B25] International Organization for Standardization [ISO] (1998). *Fruit and Vegetable Products - Determination of Titratable Acidity.* Geneva: ISO.

[B26] Jin X. Chen W. Chen H. Chen W. Zhong Q. (2019). Combination of *Lactobacillus plantarum* and *Saccharomyces cerevisiae* DV10 as starter culture to produce mango slurry: microbiological, chemical parameters and antioxidant activity. *Molecules.* 24:4349. 10.3390/molecules24234349 31795169 PMC6930673

[B27] Kadam S. Prabhasankar P. (2010). Marine foods as functional ingredients in bakery and pasta products. *Food Res. Int.* 43 1975–1980. 10.1016/j.foodres.2010.06.007

[B28] Kuda T. Nemoto M. Kawahara M. Oshio S. Takahashi H. Kimura B. (2015). Induction of the superoxide anion radical scavenging capacity of dried ‘*funori*’ *Gloiopeltis furcata* by *Lactobacillus plantarum* S-SU1 fermentation. *Food Funct.* 6 2535–2541. 10.1039/C5FO00668F 26110834

[B29] Liang Y.-R. Zhang L. Lu J. (2005). A study on chemical estimation of Pu-erh tea quality. *J. Sci. Food Agric.* 85 381–390. 10.1002/jsfa.1857

[B30] McDonald S. Prenzler P. Antolovich M. Robards K. (2001). Phenolic content and anti-oxidant activity of olive extract. *Food Chem.* 73 73–84. 10.1016/S0308-8146(00)00288-0

[B31] Nami Y. Bakhshayesh R. V. Manafi M. Hejazi M. A. (2019). Hypocholesterolaemic activity of a novel autochthonous potential probiotic *Lactobacillus plantarum* ys5 isolated from yogurt. *LWT Food Sci. Technol.* 111 876–882. 10.1016/j.lwt.2019.05.057

[B32] Nan B. Liu Y. You Y. Li W. Fan J. Wang Y. (2018). Protective effects of enhanced minor ginsenosides in *Lactobacillus* fermentum KP-3-fermented ginseng in mice fed a high fat diet. *Food Funct.* 9 6020–6028. 10.1039/C8FO01056K 30397690

[B33] Papanna S. Halami P. M. Nm S. (2012). Potential of marine lactic acid bacteria to ferment *Sargassum* sp. for enhanced anticoagulant and antioxidant properties. *J. Appl. Microbiol.* 114 96–107. 10.1111/jam.12023 23020529

[B34] Pyo Y.-H. Seong K.-S. (2009). Hypolipidemic effects of *Monascus*-fermented soybean extracts in rats fed a high-fat and -cholesterol diet. *J. Agric. Food Chem.* 57 8617–8622. 10.1021/jf901878c 19697921

[B35] Rafiquzzaman S. M. Kong I. S. Kim J. M. (2015). Enhancement of antioxidant activity, total phenolic and flavonoid content of *Saccharina japonica* by submerged fermentation with *Aspergillus oryzae*. *KSBB J.* 30 27–32. 10.7841/ksbbj.2015.30.1.27

[B36] Russo P. Englezos V. Capozzi V. Pollon M. Segade S. Rantsiou K. (2020). Effect of mixed fermentations with *Starmerella bacillaris* and *Saccharomyces cerevisiae* on management of malolactic fermentation. *Food Res. Int.* 134:109246. 10.1016/j.foodres.2020.109246 32517918

[B37] Saikia D. Manhar A. K. Deka B. Roy R. Gupta K. Namsa N. D. (2018). Hypocholesterolemic activity of indigenous probiotic isolate *Saccharomyces cerevisiae* ARDMC1 in a rat model. *J. Food Drug Anal.* 26 154–162. 10.1016/j.jfda.2016.12.017 29389551 PMC9332649

[B38] Suraiya S. Lee J. M. Cho H. J. Jang W. J. Kim D.-G. Kim Y.-O. (2018). *Monascus* spp. fermented brown seaweeds extracts enhance bio-functional activities. *Food Biosci.* 21 90–99. 10.1016/j.fbio.2017.12.005

[B39] Talawar S. Harohally N. Ramakrishna C. Gurusiddaiah S. (2017). Development of wheat bran oil concentrates rich in bioactives with antioxidant and hypolipidemic properties. *J. Agric. Food Chem.* 65 9838–9848. 10.1021/acs.jafc.7b03440 29047281

[B40] Tan I. S. Lee K. T. (2015). Solid acid catalysts pretreatment and enzymatic hydrolysis of macroalgae cellulosic residue for the production of bioethanol. *Carbohydr. Polym.* 124 311–321. 10.1016/j.carbpol.2015.02.046 25839825

[B41] Wang D. Zhao Y. Jiao Y. Yu L. Yang S. Yang X. (2012). Antioxidative and hepatoprotective effects of the polysaccharides from *Zizyphus jujube* cv. *Shaanbeitanzao*. *Carbohyd. Polym.* 88 1453–1459. 10.1016/j.carbpol.2012.02.046

[B42] Wang G. Huang W. Xia Y. Xiong Z. Ai L. (2019). Cholesterol-lowering potentials of *Lactobacillus* strain overexpression of bile salt hydrolase on high cholesterol diet-induced hypercholesterolemic mice. *Food Funct.* 10 1684–1695. 10.1039/C8FO02181C 30839966

[B43] Zhou K. Xia W. Zhang C. Yu L. (2006). In vitro binding of bile acids and triglycerides by selected chitosan preparations and their physico-chemical properties. *LWT Food Sci. Technol.* 39 1087–1092. 10.1016/j.lwt.2005.07.009

