# Supplementary figures and images for: Hypolipidemic Effects of Fermented Seaweed Extracts by Saccharomyces cerevisiae and Lactiplantibacillus plantarum
Source: Front Microbiol. 2021 Nov 12;12:772585. doi: 10.3389/fmicb.2021.772585 (PMC8633411; doi:10.3389/fmicb.2021.772585)

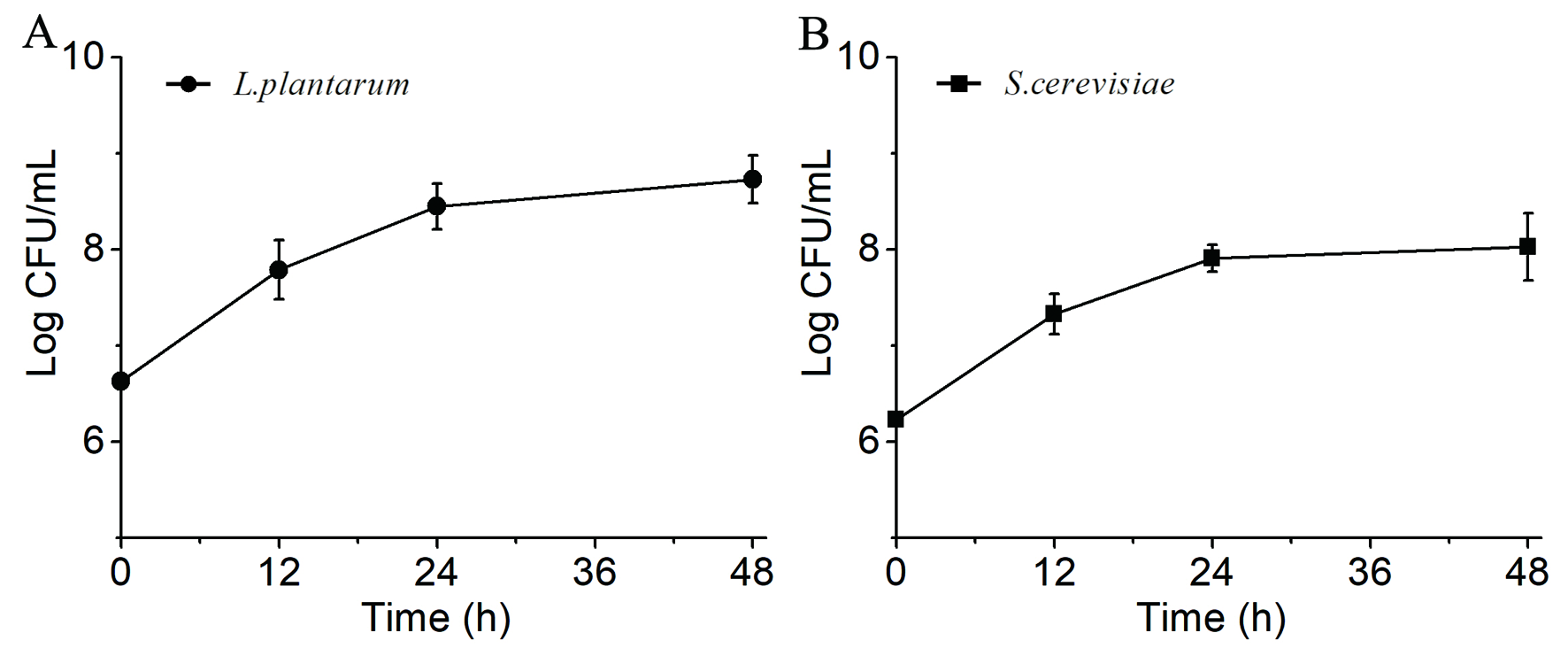

Supplement: Supplementary Figure 1 — Cell growth of L. plantarum (A) and S. cerevisiae (B) during single culture fermentations. [file Image_1.TIF]
